# Investigations of the Effects of Geometric Imperfections on the Nonlinear Static and Dynamic Behavior of Capacitive Micomachined Ultrasonic Transducers

**DOI:** 10.3390/mi9110575

**Published:** 2018-11-05

**Authors:** Aymen Jallouli, Najib Kacem, Joseph Lardies

**Affiliations:** Department of Applied Mechanics, FEMTO-ST Institute, UMR 6174, CNRS/UFC/ENSMM/UTBM, Univ. Bourgogne Franche-Comté, F-25000 Besançon, France; aymen.jallouli@femto-st.fr (A.J.); joseph.lardies@univ-fcomte.fr (J.L.)

**Keywords:** CMUT, nonlinear dynamics, geometric imperfection, von Kármán plate theory, differential quadrature method

## Abstract

In order to investigate the effects of geometric imperfections on the static and dynamic behavior of capacitive micomachined ultrasonic transducers (CMUTs), the governing equations of motion of a circular microplate with initial defection have been derived using the von Kármán plate theory while taking into account the mechanical and electrostatic nonlinearities. The partial differential equations are discretized using the differential quadrature method (DQM) and the resulting coupled nonlinear ordinary differential equations (ODEs) are solved using the harmonic balance method (HBM) coupled with the asymptotic numerical method (ANM). It is shown that the initial deflection has an impact on the static behavior of the CMUT by increasing its pull-in voltage up to 45%. Moreover, the dynamic behavior is affected by the initial deflection, enabling an increase in the resonance frequencies and the bistability domain and leading to a change of the frequency response from softening to hardening. This model allows MEMS designers to predict the nonlinear behavior of imperfect CMUT and tune its bifurcation topology in order to enhance its performances in terms of bandwidth and generated acoustic power while driving the microplate up to 80% beyond its critical amplitude.

## 1. Introduction

Microresonators have been used in several applications such as mass sensing [1], micropumps [2], gas sensors [3], gyroscopes [4] and accelerometers [5]. These microsystems are commonly fabricated with an initial deflection due to the mechanical stress caused by the fabrication process.

One of the most famous micro-systems that have been developed is the capacitive micromachined ultrasonic transducer (CMUT), which is a flexible microplate that can vibrate under an applied voltage. CMUTs have been used as transducers by emitting ultrasonic waves, also as a sensors to capture ultrasounds. Despite the excessive use of piezoelectric transducers in industrial applications, the capacitive micro-machined ultrasonic transducers (CMUTs) are more advantageous than the piezoelectric ones. First they can be fabricated using micromachining techniques [6,7,8] with tight parameter specifications and with complex shapes (circular, square, hexagonal...) [9,10,11], which makes them operate in different applications. Batch fabrication enables the micromachining of large numbers of CMUTs at the same time with good uniformity. Another important feature of CMUTs is their compatibility with integrated circuit (IC) technology. Compared to piezoelectric transducers, CMUTs have a larger bandwidth, which can result in good sensitivity (when the device is operating in the receive mode), better transmission (in the case of transmitting mode) and low noise [12].

However, the microfabrication techniques reveal several imperfections such as stress gradients [13] and initially curved shapes [14]. Even a small initial deflection, with respect to the dimensions of the resonator, has a critical effect on its static and dynamic behavior. These effects have been studied on several microresonators; for instance, the static response of a microbeam has been investigated by Ouakad et al. [15,16]. In the case of the downward initial deflection, the increase of the initial deflection leads to a decrease in the pull-in voltage due to the fact that the microbeam becomes closer to the bottom electrode. The upward initial deflection results in a snap through behavior [17,18] where the initial deflection increases the snap through voltage and decreases the pull-in voltage.

The initial imperfection also has a major effect on the resonance frequencies of a resonator. An increase of the initial deflection leads to a divergence of the odd resonance frequencies also known as frequency veering [19,20,21,22]. This phenomenon was reported in several studies such as the effect of varying the ratio of the side lengths of a rectangular plate [23] and the effect of varying the sagging levels on cables [24].

The initial deflection dose not only have an effect on the static behavior of microsystems but also affects nonlinear dynamic behavior. Ruzziconi et al. [25,26] developed a nonlinear model for a clamped microbeam obtained using Galerkin method. With only two degrees of freedom, the numerical model is able to predict with satisfactory accuracy the nonlinear dynamic behavior of the microbeam, since it correctly simulates both the primary and the 1/3 superharmonic resonances. The dynamic pull-in and dynamic snap-through of an arch microbeam was also investigated by Younis et al. [15] by exciting the microresonator with high DC and AC voltages around the fundamental resonance frequency. The increase in the shallow arch has two effects on the nonlinear behavior of the microbeam: first it reduces its vibration amplitude due to the increase of the distance between the two electrodes; and second it reduces the hardening behavior and increases its softening behavior. Experimental investigation was also conducted on the microstructure around the first and the third resonance frequency [27,28]. For low DC and AC voltages, the microsystem has a softening behavior at the first resonance frequency and a hardening behavior at the third resonance frequency. The experimental tests showed that with high voltages, we can obtain dynamic snap-through cross-well motions. Also, the microbeam can exhibit large oscillations of a continuous band of snap-through motion between the first primary resonance regime and the super-harmonic resonance regime. These characteristics of arches resonators were used in developing bandpass filters [29], low-powered switches [30] and logic devices [31].

The effect of initial imperfection was also studied for the case of microplate. Saghir et al. [32,33] derived the equation of motion of a rectangular microplate excited with an electrostatic force. The governing equations, derived using the von Kármán plate theory, are transformed into a Reduced Order Model (ROM) using the Galerkin discretization procedure. The static and the dynamic behavior of the microsystem were studied experimentally and the obtained results were compared to the numerical results obtained with the developed model. Near primary resonance, the microplate has a hardening type behavior when the excitation force is low and the increase in the AC voltage increases the gap between the two stable solutions. The dynamic behavior of the microresonator was also investigated at ω/3 super-harmonic excitation. In this case, the increase in the AC voltage does not only increase the vibration amplitude but also changes the nonlinear dynamic behavior from hardening to softening.

Several studies have been conducted on the nonlinear behavior of rectangular plate with imperfection at the macroscale. Celep et al. [34,35] investigated the effect of an initial imperfection on the static and dynamic behavior of a rectangular plate subjected to four different boundary conditions. Chena et al. [36] derived the nonlinear differential equation of the rectangular plate with initial imperfection and under arbitrary initial stress. The governing equations are discretized using the Galerkin procedure and the partial differential equations are transformed into a set of ordinary differential equations. The obtained equations were solved using the Runge-Kutta method to obtain the linear and nonlinear vibration frequencies.

Medina et al. [37,38] developed a reduced order model which has been validated using finite element analysis in order to study the axisymmetric snap-through of an initially curved circular plate subjected to an electrostatic actuation. The analysis revealed that due to the nonlinearity of the electrostatic force, the snap-through occurs at a lower displacement compared to the one induced by mechanical loads. For the case of CMUTs, few studies have been conducted on nonlinear behavior. Vogl et al. [39,40] used the von Kármán plate theory and the Galerkin method to determine the nonlinear differential equations of a circular CMUT. These equations have been solved using the method of multiple scales [41]. This analytical method is used to determine the nonlinear frequency and force responses of the CMUT around the first resonance frequency. The microsystem can display a softening or a hardening behavior depending on the applied DC voltage.

The nonlinear behavior of circular plates was also investigated at the macroscale by Touzé and Thomas [42,43]. The circular plate, with free edge boundary condition, was forced with a frequency around the asymmetric mode by taking into account the effects of damping and small geometric imperfection. The nonlinear frequency response of the system can exhibit different nonlinear behavior, softening or hardening, depending on the design parameters. From these solutions they showed that traveling waves are generic when coupled-mode solutions arise. The analytical model has been validated with respect to experimental results obtained by measuring the deflections of an antinode of each preferential configuration. By estimating the input parameter of the system, the developed model was able to predict the nonlinear phenomena of the plate.

The differential quadrature method is a numerical technique that has been used for discretization of a mathematical problem. It was derived by Richard Bellman in the early 1970s [44]. This method has been employed as an alternative technique for finite difference method and FEM. We can mention the work of Najar et al. [45] in modeling microbeams. The static and dynamic behavior of the microbeam were studied by discretizing the partial differential equation using DQM. This method is accurate and efficient. With only nine grid points, the error in the pull-in voltage is about 0.2%. The Generalized Differential Quadrature Finite Element Method (GDQFEM) was also used to solve the static and dynamic behavior of plates with complex shapes [46].

In this paper, the nonlinear behavior of initially curved circular CMUTs is modeled. The partial differential equations of the upper microplate of the CMUT are derived based on the von Kármán plate theory, while including the initial curvature, the geometric and the electrostatic nonlinearities. First, the differential quadrature method (DQM) is used to transform the governing equations into a system of ordinary differential equations with respect to time. Then, the time derivatives are dropped and the resulting coupled algebraic equations are solved in order to investigate the static behavior of the CMUT with respect to the DC voltage and the initial deflection. The obtained results are validated with respect to the experimental data. Moreover, the nonlinear frequency and force responses are investigated by solving the nonlinear differential equations using the harmonic balance method (HBM) coupled with the asymptotic numerical method (ANM). Finally, the effects of the actuation voltages and the initial deflection on the dynamic behavior of the CMUT are numerically investigated.

## 2. Mathematical Model

**Figure 1:**
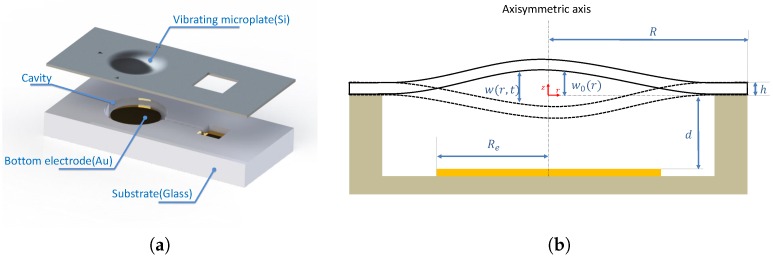
(**a**) Exploded view of a Capacitive Micomachined Ultrasonic Transducer (CMUT). (**b**) A cross section of a circular electrostatic thin microplate.

In this section, we consider a circular microplate with a uniform cross-section *h* and a homogeneous material with a density ρ and a Young’s modulus *E*, as shown in Figure 1. The vibrating electrode is modeled as a perfectly clamped circular microplate with radius *R* excited with an electric voltage $V\left(t\right)=V_{dc}+V_{ac}\;cos\left(\omega t\right)$.

The bottom electrode, with radius $R_{e}$, is placed below the microplate at a distance *d*. We suppose that the microplate is subjected to an axisymetric initial deflection $w_{0}\left(r\right)$ defined as follows:

(1) $$w_{0}\left(r\right)=w_{\max}\left[1-{\left(\frac{r}{R}\right)}^{2}+2{\left(\frac{r}{R}\right)}^{2}\ln\left(\frac{r}{R}\right)\right]$$

where $w_{\max}$ represent the initial deflection of the membrane at the center. We have chosen this expression of the initial deflection because it satisfies the boundary conditions of a clamped circular microplate and also it is a good approximation to the initial deflection determined experimentally [47].

The microplate equations of motion are derived from the von Kármán plate theory. Luan and Robbins [48] proved that, at the microscale, the continuum mechanics theory is still valid. We consider the following hypothesis of the von Kármán plate theory [49]:

- The material of the plate is elastic, homogeneous, and isotropic.
- A straight line (filament), initially normal to the midplane, remains straight and normal to the surface during the deformation.
- The stress normal to the midplane, $\sigma_{z}$, is small compared to the other stress components and may be neglected in the stress-strain relations.

### 2.1. Problem Formulation

We follow the work of Nayfeh [50] to derive the equations of motion for an axisymmetric circular plate. We denote u(r,t) and w(r,t) the displacement component of a point in the plate with respect to the r,θ,z coordinate system. The displacement vector $\vec{D}$ is given by:

(2) $$\vec{D}=\left(\begin{array}{c} u-zw_{,r}\\ 0\\ w \end{array}\right)$$

where $w_{,r}$ is the derivative of *w* with respect to *r*. The von Kármán nonlinear strains for a plate in large displacement are given by:

(3) $$\left\{\begin{array}{c} \varepsilon_{rr}=e_{1}-zw_{,rr}+\varepsilon_{0}\\ \varepsilon_{\theta \theta }=e_{2}-z\frac{w_{,r}}{r}+\varepsilon_{0}\\ \varepsilon_{r\theta }=\varepsilon_{zz}=\varepsilon_{z\theta }=\varepsilon_{zr}=0 \end{array}\right.$$

where $w_{,rr}$ is the second derivative of *w* with respect to *r*. $\varepsilon_{0}$ represents the initial strain due to the residual stress $N_{0}$. $e_{1}$ and $e_{2}$ are the midplane strain components and are given as:

(4) $$\begin{array}{cc} e_{1} & =u_{,r}+\frac{1}{2}w_{,r}^{2}+w_{,r}w_{0,r}\\ e_{2} & =\frac{u}{r}\\ \varepsilon_{0} & =N_{0}\frac{\left(1-\nu \right)}{Eh} \end{array}$$

where ν is the Poisson’s ratio and $w_{0,r}$ is the first derivative of the initial deflection $w_{0}$ with respect to *r*. In order to obtain the equations of motion and the boundary conditions, we use the extended Hamilton principle which can be written as:

(5) $$\int_{t_{1}}^{t_{2}}\left(\delta T-\delta \Pi +\delta W_{e}+\delta W_{nc}\right)dt=0$$

where $\delta W_{nc}$, δΠ, δT and $\delta W_{e}$ denote the variations of the nonconservative, kinetic, elastic and electrostatic potential energies ($W_{nc}$, Π, *T* and $W_{e}$), expressed, respectively, as:

(6) $$\delta \Pi =\int_{z}\int_{A}\left(\sigma_{rr}\delta \varepsilon_{rr}+\sigma_{\theta \theta }\delta \varepsilon_{\theta \theta }\right)dAdz$$

(7) $$\delta T=-\int_{z}\int_{A}\rho \ddot{\vec{D}}\delta \vec{D}dAdz$$

The variation of the electrostatic energy for a parallel plate capacitor with an initial deflection $w_{0}$ is defined as:

(8) $$\delta W_{e}=\int_{A}\frac{\epsilon_{0}V^{2}\left(t\right)}{2\left(d+w_{0}-w\right)}\delta wdA$$

where A is the undeformed area of the reference plane. σ and ε are the Jaumann stresses and strains, respectively. $\epsilon_{0}$ is the dielectric constant of the plate material and V(t) is the applied voltage. The second time derivative and the spatial variation of the displacement vector can be written as:

(9) $$\begin{array}{ccccccccc} \ddot{\vec{D}} & =\left(\begin{array}{c} \ddot{u}-z{\ddot{w}}_{,r}\\ 0\\ \ddot{w} \end{array}\right) & & & & & & \delta \vec{D} & =\left(\begin{array}{c} \delta u-z\delta w_{,r}\\ 0\\ \delta w \end{array}\right) \end{array}$$

Substituting Equation (9) into Equation (7) and integrating over the thickness $z\in \left[-h/2,h/2\right]$, yields:

(10) $$\delta T=-\int_{A}\left(\rho h\left(\ddot{u}\delta u+\ddot{w}\delta w\right)+\frac{\rho h^{3}}{12}{\ddot{w}}_{r}\delta w_{,r}\right)dA$$

Substituting Equation (3) into Equation (6), yields

(11) $$\delta \Pi =\int_{z}\int_{A}\left(\sigma_{rr}\left(\delta u_{,r}+\delta w_{,r}\left(w_{,r}+w_{0,r}\right)-z\delta w_{,rr}\right)+\sigma_{\theta \theta }\left(\frac{\delta u}{r}-z\frac{\delta w_{,r}}{r}\right)\right)dAdz$$

Integrating over the thickness $z\in \left[-h/2,h/2\right]$, we obtain

(12) $$\delta \Pi =\int_{A}\left(N_{rr}\left(\delta u_{,r}+\delta w_{,r}\left(w_{,r}+w_{0,r}\right)\right)-M_{rr}\delta w_{,rr}+N_{\theta \theta }\frac{\delta u}{r}-M_{\theta \theta }\frac{\delta w_{,r}}{r}\right)dA$$

where $M_{\theta \theta }$ and $M_{rr}$ are the plate moments. $N_{\theta \theta }$ and $N_{rr}$ are the plate stress resultants:

(13) $$\left\{N_{rr},N_{\theta \theta }\right\}=\int_{z}\left\{\sigma_{rr},\sigma_{\theta \theta }\right\}dz$$

(14) $$\left\{M_{rr},M_{\theta \theta }\right\}=\int_{z}z\left\{\sigma_{rr},\sigma_{\theta \theta }\right\}dz$$

We perform partial integration for the following terms:

(15a) $$\int_{A}N_{rr}\delta u_{,r}dA=2\pi \left(-\int_{r}\left(N_{rr}+rN_{rr,r}\right)\delta udr+{\left[rN_{rr}\delta u\right]}_{0}^{R}\right)$$

(15b) $$\begin{array}{c} \int_{A}\left(N_{rr}\left(w_{,r}+w_{0,r}\right)-\frac{M_{\theta \theta }}{r}\right)\delta w_{,r}dA=2\pi (-\int_{r}(N_{rr}\left(w_{,r}+w_{0,r}\right)\\ +rN_{rr,r}\left(w_{,r}+w_{0,r}\right)+rN_{rr}\left(w_{,rr}+w_{0,rr}\right)-M_{\theta \theta ,r})\delta wdr+{\left[\left(rN_{rr}\left(w_{,r}+w_{0,r}\right)-M_{\theta \theta }\right)\delta w\right]}_{0}^{R}) \end{array}$$

(15c) $$-\int_{A}M_{rr}\delta w_{,rr}dA=2\pi \left(-\int_{A}\left(2M_{rr,r}+rM_{rr,rr}\right)\delta wdr-{\left[rM_{rr}\delta w_{,r}\right]}_{0}^{R}+{\left[\left(M_{rr}+rM_{rr,r}\right)\delta w\right]}_{0}^{R}\right)$$

where $w_{0,rr}$ is the second derivative of the initial deflection $w_{0}$ with respect to *r*. Substituting (15) into (12), we obtain:

(16) $$\begin{array}{cc} \delta \Pi = & 2\pi (-\int_{r}((N_{rr}\left(w_{,r}+w_{0,r}\right)+rN_{rr,r}\left(w_{,r}+w_{0,r}\right)+rN_{rr}\left(w_{,rr}+w_{0,rr}\right)\\ & -M_{\theta \theta ,r}+2M_{rr,r}+rM_{rr,rr})\delta w+\left(N_{rr}+rN_{rr,r}-N_{\theta \theta }\right)\delta u)dr+{\left[rN_{\mathrm{rr}}\delta u\right]}_{0}^{R}\\ & +{\left[\left(rN_{rr}\left(w_{,r}+w_{0,r}\right)-M_{\theta \theta }\right)\delta w\right]}_{0}^{R}-{\left[rM_{rr}\delta w_{,r}\right]}_{0}^{R}+{\left[\left(M_{rr}+rM_{rr,r}\right)\delta w\right]}_{0}^{R}) \end{array}$$

Substituting (16), (10) and (8) into (5) and setting each of the coefficients of δu and δw equal to zero, we obtain

(17) $$\begin{array}{c} N_{rr}\left(w_{,r}+w_{0,r}\right)+rN_{rr,r}\left(w_{,r}+w_{0,r}\right)+rN_{rr}\left(w_{,rr}+w_{0,rr}\right)\\ -M_{\theta \theta ,r}+2M_{rr,r}+rM_{rr,rr}+r\rho h\ddot{w}-r\frac{\epsilon_{0}V^{2}\left(t\right)}{2\left(d+w_{0}-w\right)}=0 \end{array}$$

(18) $$N_{rr}+rN_{rr,r}-N_{\theta \theta }+r\rho h\ddot{u}=0$$

where $N_{rr,r}$$M_{\theta \theta ,r}$ and $M_{rr,r}$ are the first derivatives of $N_{rr}$, $M_{\theta \theta }$ and $M_{rr}$ with respect to *r*. $M_{rr,rr}$ is the second derivative of $M_{rr}$ with respect to *r*. The corresponding boundary conditions at r=0 and r=R are defined as:

(19) $$\begin{array}{ccccccccccc} rN_{rr} & =0 & & & & \mathrm{or} & & & & \delta u & =0\\ rN_{rr}w_{,r}-M_{\theta \theta }+M_{rr}+rM_{rr,r} & =0 & & & & \mathrm{or} & & & & \delta w & =0\\ rM_{rr} & =0 & & & & \mathrm{or} & & & & \delta w_{,r} & =0 \end{array}$$

### 2.2. Dimensional Equations of Motion

For thin plates, we adopt the plane stress assumption. The stress-strain relations for an linear elastic material can be stated as [49]:

(20) $$\left\{\begin{array}{c} \sigma_{rr}\\ \sigma_{\theta \theta }\\ \sigma_{r\theta } \end{array}\right\}=\frac{E}{1-\nu^{2}}\left[\begin{array}{ccc} 1 & \nu & 0\\ \nu & 1 & 0\\ 0 & 0 & \frac{1-\nu }{2} \end{array}\right]\left\{\begin{array}{c} \varepsilon_{rr}\\ \varepsilon_{\theta \theta }\\ \varepsilon_{r\theta } \end{array}\right\}$$

Therefore

(21) $$\begin{array}{cc} \sigma_{rr} & =\frac{E}{1-\nu^{2}}\left(\varepsilon_{rr}+\nu \varepsilon_{\theta \theta }\right)\\ \sigma_{\theta \theta } & =\frac{E}{1-\nu^{2}}\left(\varepsilon_{\theta \theta }+\nu \varepsilon_{rr}\right)\\ \sigma_{r\theta } & =G\varepsilon_{r\theta } \end{array}$$

where $G=\frac{E}{2(1+\nu )}$ is the shear modulus. Substituting Equation (21) into Equations (13) and (14) and performing the integration yields

(22) $$\begin{array}{cc} N_{rr} & =\frac{Eh}{1-\nu^{2}}\left(u_{,r}+\frac{1}{2}w_{,r}^{2}+w_{,r}w_{0,r}+\varepsilon_{0}+\nu \left(\frac{u}{r}+\varepsilon_{0}\right)\right)\\ N_{\theta \theta } & =\frac{Eh}{1-\nu^{2}}\left(\frac{u}{r}+\varepsilon_{0}+\nu \left(u_{,r}+\frac{1}{2}w_{,r}^{2}+w_{,r}w_{0,r}+\varepsilon_{0}\right)\right)\\ M_{rr} & =-\frac{Eh^{3}}{12\left(1-\nu^{2}\right)}\left(w_{,rr}+\nu \frac{w_{,r}}{r}\right)\\ M_{\theta \theta } & =-\frac{Eh^{3}}{12\left(1-\nu^{2}\right)}\left(\frac{w_{,r}}{r}+\nu w_{,rr}\right) \end{array}$$

By substituting Equations (22) into the Equations of motion (17) and (18), neglecting the inplane and rotatory inertia terms (since they have negligible effect on the transverse motion because the transverse natural frequencies are negligible to the inplane natural frequency [49]) and adding the damping force, the governing equations can be written as:

(23) $$\begin{array}{cc} \frac{\rho h^{2}}{D}\ddot{w} & +\frac{h^{2}}{D}f_{d}+\nabla^{4}w=\frac{N_{0}}{D}\left(\frac{1}{r}\left(w_{,r}+w_{0,r}\right)+\left(w_{,rr}+w_{0,rr}\right)\right)+\frac{\epsilon_{0}V^{2}\left(t\right)}{2D{\left(d-w+w_{0}\right)}^{2}}\\ & +\frac{12}{h^{2}}[\frac{1}{r}u_{,r}\left(w_{,r}+w_{0,r}\right)+u_{,rr}\left(w_{,r}+w_{0,r}\right)+u_{,r}\left(w_{,rr}+w_{0,rr}\right)+\frac{1}{2r}{\left(w_{,r}\right)}^{2}\left(w_{,r}+w_{0,r}\right)\\ & +w_{,r}\left(w_{,r}+w_{0,r}\right)\left(\frac{1}{r}w_{0,r}+w_{0,rr}\right)+\frac{1}{2}{\left(w_{,r}\right)}^{2}\left(w_{,rr}+w_{0,rr}\right)+w_{,r}w_{0,r}\left(w_{,rr}+w_{0,rr}\right)\\ & +w_{,rr}{\left(w_{,r}+w_{0,r}\right)}^{2}+\frac{\nu }{r}\left(u_{,r}\left(w_{,r}+w_{0,r}\right)+u\left(w_{,rr}+w_{0,rr}\right)\right)] \end{array}$$

(24) $$u_{,rr}+\frac{1}{r}u_{,r}-\frac{u}{r^{2}}=-\frac{1-\nu }{2r}{\left(w_{,r}\right)}^{2}-w_{,r}w_{,rr}$$

This system of differential equations takes into consideration the effects of both the initial deflection and the residual stress. The last term between two brackets in Equation (23) represents the mid-plane stretching effects which are caused by the large displacement of the microplate. The axisymmetric boundary conditions are defined with a horizontal tangent at r=0 and a lateral displacement equal to zero u=0. For the clamped boundary condition (r=R), the in-plane and the out-of-plane displacements *u* and *w* are equal to zero. Therefore the boundary conditions of the clamped circular microplate are:

(25) $$\begin{array}{cccccccccccc} \mathrm{At}\; & \;r=0: & & & & w_{,r} & =0 & & & & u & =0\\ \mathrm{At}\; & \;r=R: & & & & w_{,r} & =w=0 & & & & u & =0 \end{array}$$

where $\nabla^{4}$ is the bi-harmonic operator expressed as $\nabla^{4}={\left(\frac{\partial^{2}}{\partial r^{2}}+\frac{1}{r}\frac{\partial }{\partial r}\right)}^{2}$, $D=\frac{Eh^{3}}{12\left(1-\nu^{2}\right)}$ is the plate flexural rigidity and $f_{d}=c\dot{w}$ is the viscous damping force. The electric forcing term in Equation (23) is a parallel-plate approximation to the capacitance where the fringing field effect is ignored for a small aspect ratio d≪R of the capacitor [51].

### 2.3. Nondimensional Equations of Motion

In order to reduce the complexity of the physical parameters, we rewrite Equations (23) and (24) in terms of nondimensional variables, which are defined as follow:

(26) $$\begin{array}{cccccccccccc} \tilde{r} & =Rr; & & & & \tilde{u} & =du; & & & & \tilde{w} & =dw\\ \tilde{c} & =\frac{{\left(D\rho h\right)}^{1/2}}{R^{2}}c; & & & & \gamma & =\frac{12Rd}{h^{2}}; & & & & \beta & =\frac{d}{R}\\ {\tilde{N}}_{0} & =\frac{D}{R^{2}}N_{0}; & & & & \tilde{t} & =Tt=R^{2}{\left(\frac{\rho h}{D}\right)}^{1/2}t; & & & & \alpha_{e} & =\frac{\varepsilon_{0}R^{4}}{2Dd^{3}} \end{array}$$

Substituting these definitions into (23) and (24) and dropping the tilde (∼) in the result, we get:

(27) $$\ddot{w}+c\dot{w}+\nabla^{4}w=N_{0}\left(\frac{1}{r}\left(w_{,r}+w_{0,r}\right)+\left(w_{,rr}+w_{0,rr}\right)\right)+\alpha_{e}\frac{V^{2}\left(t\right)}{{\left(1-w+w_{0}\right)}^{2}}+\gamma \hat{\Gamma }\left(w,u,w_{0}\right)$$

(28) $$u_{,rr}+\frac{1}{r}u_{,r}-\frac{u}{r^{2}}=-\frac{1-\nu }{2r}\left({\left(w_{,r}\right)}^{2}+2w_{,r}w_{0,r}\right)-w_{,rr}\left(w_{,r}+w_{0,r}\right)-w_{,r}w_{0,rr}$$

where Γ is defined as:

(29) $$\begin{array}{cc} \hat{\Gamma }\left(w,u,w_{0}\right) & =\frac{1}{r}u_{,r}\left(w_{,r}+w_{0,r}\right)+u_{,rr}\left(w_{,r}+w_{0,r}\right)+u_{,r}\left(w_{,rr}+w_{0,rr}\right)\\ & +\beta (\frac{1}{2r}{\left(w_{,r}\right)}^{2}\left(w_{,r}+w_{0,r}\right)+w_{,rr}{\left(w_{,r}+w_{0,r}\right)}^{2}+w_{,r}\left(w_{,r}+w_{0,r}\right)\left(\frac{1}{r}w_{0,r}+w_{0,rr}\right)\\ & +\frac{1}{2}{\left(w_{,r}\right)}^{2}\left(w_{,rr}+w_{0,rr}\right)+w_{,r}w_{0,r}\left(w_{,rr}+w_{0,rr}\right))+\frac{\nu }{r}\left(u_{,r}\left(w_{,r}+w_{0,r}\right)+u\left(w_{,rr}+w_{0,rr}\right)\right) \end{array}$$

where ${\tilde{w}}_{0}=d\;w_{0}$ and the tilde (∼) represents the dimensional quantities. For convenience, we rewrite the boundary conditions in its nondimensional form:

(30) $$\begin{array}{cccccc} \mathrm{At}\; & \;r=0: & w_{,r} & =0 & u & =0\\ \mathrm{At}\; & \;r=1: & w_{,r} & =w=0 & u & =0 \end{array}$$

## 3. Discretization

### 3.1. Differential Quadrature Method

In order to solve the equation of motion of the system, we reduce the PDEs into a set of ODEs. One of the methods that has been used in the literature is the differential quadratic method. The aim of this method is to discretize the space domain into a grid of sampling points and approximating the derivative with respect to *r* as a weighted sum of the function at the grid points. To this end, the derivatives can be written as:

(31) $$\begin{array}{ccccccccc} {\left[\frac{\partial^{k}w}{\partial r^{k}}\right]}_{r=r_{i}} & =\sum_{j=1}^{n}A_{ij}^{\left(k\right)}w_{j} & & & & & & {\left[\frac{\partial^{k}u}{\partial r^{k}}\right]}_{r=r_{i}} & =\sum_{j=1}^{n}A_{ij}^{\left(k\right)}u_{j} \end{array}$$

where $r_{i}$ are the grid points defined by Chebyshev-Gauss-Lobatto [52]:

(32) $$r_{i}=\frac{1}{2}\left(1-\cos\left(\frac{\left(i-1\right)}{\left(n-1\right)}\pi \right)\right)$$

$u_{i}$, $w_{i}$ are the in-plane and out-of-plane displacements of plate at the grid point $r=r_{i}$. $A_{ij}^{\left(k\right)}$ are the DQM weighting coefficients of the *k*th order derivative which are given by [53,54,55]:

(33) $$\begin{array}{ccccc} A_{ij}^{\left(1\right)} & =\frac{\prod_{v=1;v\neq i}^{n}\left(r_{i}-r_{v}\right)}{\left(r_{i}-r_{j}\right)\prod_{v=1;v\neq j}^{n}\left(r_{j}-r_{v}\right)} & i,j & =1,2,\cdots ,n & i\neq j\\ A_{ij}^{\left(k\right)} & =k\left(A_{ii}^{\left(k-1\right)}A_{ij}^{\left(1\right)}-\frac{A_{ij}^{\left(k-1\right)}}{\left(r_{i}-r_{j}\right)}\right) & i,j & =1,2,\cdots ,n & i\neq j\\ A_{ii}^{\left(k\right)} & =-\sum_{v=1;v\neq i}^{n}A_{iv}^{\left(k\right)} & i & =1,2,\cdots ,n \end{array}$$

where $A_{ij}^{\left(k\right)}$ are the respective weighting coefficients that depend only on the sampling points. The advantage of using this configuration is that it can converge only with a few number of grid points. Also, it reduces the computational time as a result of the properties of the weighting matrix $\left[A^{\left(k\right)}\right]$ [56,57].

### 3.2. Discretized Equations of Motion

If we apply the DQM to the equation of motion (27) and (28), we obtain:

(34) $$\begin{array}{cc} {\ddot{w}}_{i}+c{\dot{w}}_{i}+\nabla^{4}w_{i} & =N_{0}\left(\frac{1}{r_{i}}\left(\sum_{j=1}^{n}A_{ij}^{\left(1\right)}w_{j}+w_{0,r}^{i}\right)+\left(\sum_{j=1}^{n}A_{ij}^{\left(2\right)}w_{j}+w_{0,rr}^{i}\right)\right)\\ & +\alpha_{e}\frac{V^{2}\left(t\right)}{{\left(1-w_{i}+w_{0}^{i}\right)}^{2}}+\gamma {\hat{\Gamma }}_{i}\left(w_{j},u_{j},w_{0}^{i}\right);\;\;i=2,\cdots ,n-2 \end{array}$$

(35) $$\begin{array}{cc} \sum_{j=1}^{n}A_{ij}^{\left(2\right)}u_{j}+\frac{1}{r_{i}}\sum_{j=1}^{n}A_{ij}^{\left(1\right)}u_{j}-\frac{u_{i}}{r_{i}^{2}} & =-\beta (\frac{1-\nu }{2r_{i}}\left({\left(\sum_{j=1}^{n}A_{ij}^{\left(1\right)}w_{j}\right)}^{2}+2w_{0,r}^{i}\sum_{j=1}^{n}A_{ij}^{\left(1\right)}w_{j}\right)\\ & +\sum_{j=1}^{n}A_{ij}^{\left(2\right)}w_{j}\left(\sum_{j=1}^{n}A_{ij}^{\left(1\right)}w_{j}+w_{0,r}^{i}\right)+w_{0,rr}^{i}\sum_{j=1}^{n}A_{ij}^{\left(1\right)}w_{j});\;\;i=2,\cdots ,n-1 \end{array}$$

where ${\hat{\Gamma }}_{i}$ is the discretization of $\hat{\Gamma }$.

(36) $$\begin{array}{cc} {\hat{\Gamma }}_{i}\left(w_{j},u_{j},w_{0}^{i}\right) & =\frac{1}{r_{i}}\sum_{j=1}^{n}A_{ij}^{\left(1\right)}u_{j}\left(\sum_{j=1}^{n}A_{ij}^{\left(1\right)}w_{j}+w_{0,r}^{i}\right)\\ & +\sum_{j=1}^{n}A_{ij}^{\left(2\right)}u_{j}\left(\sum_{j=1}^{n}A_{ij}^{\left(1\right)}w_{j}+w_{0,r}^{i}\right)+\sum_{j=1}^{n}A_{ij}^{\left(1\right)}u_{j}\left(\sum_{j=1}^{n}A_{ij}^{\left(2\right)}w_{j}+w_{0,rr}^{i}\right)\\ & +\frac{\nu }{r_{i}}\left(\sum_{j=1}^{n}A_{ij}^{\left(1\right)}u_{j}\left(\sum_{j=1}^{n}A_{ij}^{\left(1\right)}w_{j}+w_{0,r}^{i}\right)+u_{i}\left(\sum_{j=1}^{n}A_{ij}^{\left(2\right)}w_{j}+w_{0,rr}^{i}\right)\right)\\ & +\beta (\frac{1}{2r_{i}}{\left(\sum_{j=1}^{n}A_{ij}^{\left(1\right)}w_{j}\right)}^{2}\left(\sum_{j=1}^{n}A_{ij}^{\left(1\right)}w_{j}+w_{0,r}^{i}\right)+\sum_{j=1}^{n}A_{ij}^{\left(2\right)}w_{j}{\left(\sum_{j=1}^{n}A_{ij}^{\left(1\right)}w_{j}+w_{0,r}^{i}\right)}^{2}\\ & +\sum_{j=1}^{n}A_{ij}^{\left(1\right)}w_{j}\left(\sum_{j=1}^{n}A_{ij}^{\left(1\right)}w_{j}+w_{0,r}^{i}\right)\left(\frac{1}{r_{i}}w_{0,r}^{i}+w_{0,rr}^{i}\right)\\ & +\frac{1}{2}{\left(\sum_{j=1}^{n}A_{ij}^{\left(1\right)}w_{j}\right)}^{2}\left(\sum_{j=1}^{n}A_{ij}^{\left(2\right)}w_{j}+w_{0,rr}^{i}\right)+w_{0,r}^{i}\sum_{j=1}^{n}A_{ij}^{\left(1\right)}w_{j}\left(\sum_{j=1}^{n}A_{ij}^{\left(2\right)}w_{j}+w_{0,rr}^{i}\right)) \end{array}$$

and the boundary conditions (30) can be rewritten as:

(37) $$\begin{array}{cccccc} \mathrm{At}\; & \;r=0: & \sum_{j=1}^{n}A_{1j}^{\left(1\right)}w_{j} & =0 & u_{1} & =0\\ \mathrm{At}\; & \;r=1: & \sum_{j=1}^{n}A_{nj}^{\left(1\right)}w_{j} & =w_{n}=0 & u_{n} & =0 \end{array}$$

where $w_{0}^{i}$, $w_{0,rr}^{i}$ and $w_{0,rr}^{i}$ are respectively the initial displacement and its associated first and second derivatives with respect to *r* at the $i^{th}$ grid points.

## 4. Static Response

In order to investigate the static behavior of the microplate actuated by a DC voltage, we drop the derivative with respect to time and let $w_{i}\left(t\right)$ and $u_{i}\left(t\right)$ be time independent. Therefore the equations of motion can be written as:

(38) $$\begin{array}{cc} EQ_{w}^{st}\left(i\right):\nabla^{4}w_{i}^{st} & =N_{0}\left(\frac{1}{r_{i}}\left(\sum_{j=1}^{n}A_{ij}^{\left(1\right)}w_{j}^{st}+w_{0,r}^{i}\right)+\left(\sum_{j=1}^{n}A_{ij}^{\left(2\right)}w_{j}^{st}+w_{0,rr}^{i}\right)\right)\\ & +\alpha_{e}\frac{V^{2}\left(t\right)}{{\left(1-w_{i}^{st}+w_{0}^{i}\right)}^{2}}+\gamma {\hat{\Gamma }}_{i}\left(w_{j}^{st},u_{j}^{st},w_{0}^{i}\right);\;\;i=2,\cdots ,n-2 \end{array}$$

(39) $$\begin{array}{cc} EQ_{u}^{st}\left(i\right):\sum_{j=1}^{n}A_{ij}^{\left(2\right)}u_{j}^{st} & +\frac{1}{r_{i}}\sum_{j=1}^{n}A_{ij}^{\left(1\right)}u_{j}^{st}-\frac{u_{i}^{st}}{r_{i}^{2}}=-\beta (\frac{1-\nu }{2r_{i}}\left({\left(\sum_{j=1}^{n}A_{ij}^{\left(1\right)}w_{j}^{st}\right)}^{2}+2w_{0,r}^{i}\sum_{j=1}^{n}A_{ij}^{\left(1\right)}w_{j}^{st}\right)\\ & +\sum_{j=1}^{n}A_{ij}^{\left(2\right)}w_{j}^{st}\left(\sum_{j=1}^{n}A_{ij}^{\left(1\right)}w_{j}^{st}+w_{0,r}^{i}\right)+w_{0,rr}^{i}\sum_{j=1}^{n}A_{ij}^{\left(1\right)}w_{j}^{st});\;\;i=2,\cdots ,n-1 \end{array}$$

where $w_{i}^{st}=w^{st}\left(r_{i}\right)$ and $u_{i}^{st}=u^{st}\left(r_{i}\right)$ are the static displacements of the microplate at the $i^{th}$ grid point.

### Implementation of the Arclength Method

In this section, we solve the system of Equations (38) and (39) using the arclength continuation method as defined in the Appendix A, where:

(40) $$\begin{array}{ccccc} X(s) & =\left\{w_{1}^{st}\left(s\right),w_{2}^{st}\left(s\right),\cdots ,w_{n}^{st}\left(s\right),u_{1}^{st}\left(s\right),u_{2}^{st}\left(s\right),\cdots ,u_{n}^{st}\left(s\right)\right\} & \mathrm{and} & \alpha (s) & =V_{dc}\left(s\right) \end{array}$$

and

(41) $$F\left(X\left(s\right),\alpha \left(s\right)\right)=\left\{\begin{array}{cc} \sum_{j=1}^{n}A_{1j}^{\left(1\right)}w_{j}\left(s\right)=0 & \\ EQ_{w}^{st}\left(i\right) & i=2,\cdots ,n-2\\ \sum_{j=1}^{n}A_{nj}^{\left(1\right)}w_{j}\left(s\right)=w_{n}\left(s\right)=0 & \\ u_{1}\left(s\right)=0 & \\ EQ_{u}^{st}\left(k\right) & k=2,\cdots ,n-1\\ u_{n}\left(s\right)=0 & \end{array}\right.$$

**Figure 2:**
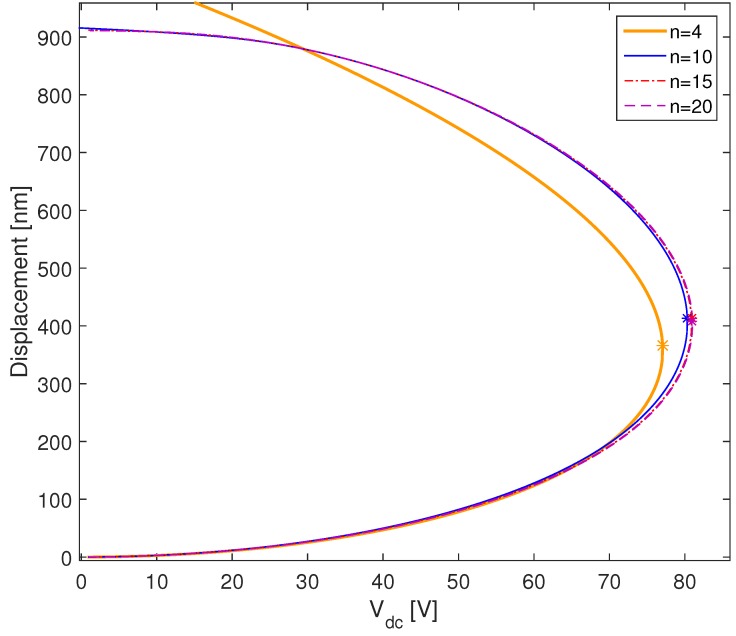
Displacement of the microplate center for different grid points *n*.

At this stage, it is crucial to check the convergence of the DQM solution. In Figure 2, we plot the total displacement of the microplate center (r=0) with respect to $V_{dc}$ voltage for different grid numbers *n*. While assuming that the residual stress $N_{0}$ is negligible, the physical parameters used in the simulation are presented in Table 1. The stars marked in this graph (∗) denote the bifurcation points where the solution changes from stable to unstable and it is called pull-in point. At these points, the slope of the curve becomes infinite. To determine the bifurcation points, we solve the following equation:

(42) $$\begin{array}{ccccccccccc} \frac{\partial w_{i}\left(s\right)}{\partial V_{dc}\left(s\right)} & =\frac{\partial w_{i}\left(s\right)}{\partial s}\frac{\partial s}{\partial V_{dc}\left(s\right)}=\infty & & & & \Rightarrow & & & & \frac{\partial V_{dc}\left(s\right)}{\partial s} & =0 \end{array}$$

At pull-in ($s=s_{pi}$), the pull-in voltage and amplitude are defined as ($V_{dc}\left(s_{pi}\right)$ , $w_{i}\left(s_{pi}\right)$). We can clearly see that the solution converges as we increase the number of grid points *n*. The advantage of using the DQM is that the solution converges with a few number of points, compared to the finite element method FEM [58].

In our case, the radius of the electrode is smaller than the radius of the membrane. Therefore, the electrostatic force is defined as a Heaviside function:

(43) $$\begin{array}{ccccccccccc} f_{e} & =\alpha_{e}\frac{V^{2}\left(t\right)}{{\left(1-w_{i}\right)}^{2}}f & & & & \mathrm{where} & & & & f & =\left\{\begin{array}{cc} 1 & r_{i}\leq \frac{R_{e}}{R}\\ 0 & r_{i}>\frac{R_{e}}{R} \end{array}\right. \end{array}$$

The problem with using this type of function is that it requires an important number of points to converge because it is not a continuous function. To increase the convergence of the DQM we use two different approximations of the Heaviside function: the first one is using a smooth logistic function defined as:

(44) $$\begin{array}{cccccccccc} f_{e} & =\alpha_{e}\frac{V^{2}\left(t\right)}{{\left(1-w_{i}\right)}^{2}}f & & & & \mathrm{where} & & & & f=\frac{1}{2}+\frac{1}{2}\tanh\left(k\left(r_{i}-\frac{R_{e}}{R}\right)\right) \end{array}$$

*k* is the steepness of the curve (k=30). The second one is by approximating the Heaviside function as the derivative of a ramp function defined as:

(45) $$f=\frac{\partial }{\partial r}\left\{\begin{array}{cc} -\frac{R_{e}}{R}+r & r\leq \frac{R_{e}}{R}\\ 0 & r>\frac{R_{e}}{R} \end{array}\right.=\sum_{j=1}^{n}A_{ij}^{\left(1\right)}\left\{\begin{array}{cc} -\frac{R_{e}}{R}+r_{j} & r_{j}\leq \frac{R_{e}}{R}\\ 0 & r_{j}>\frac{R_{e}}{R} \end{array}\right.$$

**Figure 3:**
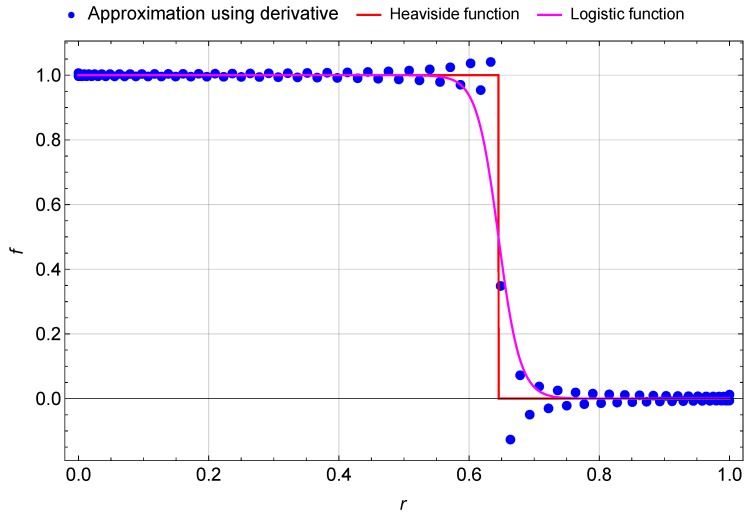
Heaviside function and its approximation.

In Figure 3 we compare the Heaviside function with its approximation. The logistic function is a good approximation of the Heaviside function except near the discontinuity point ($r=\frac{R_{e}}{R}$) where the transition is much smoother. However, for the second approximation, using the derivative of the ramp function, the solution is oscillatory especially near the singular point ($r=\frac{R_{e}}{R}$). This effect has been reported earlier by Eftekhari [59] and Jung [60] in the case of the Dirac function.

**Figure 4:**
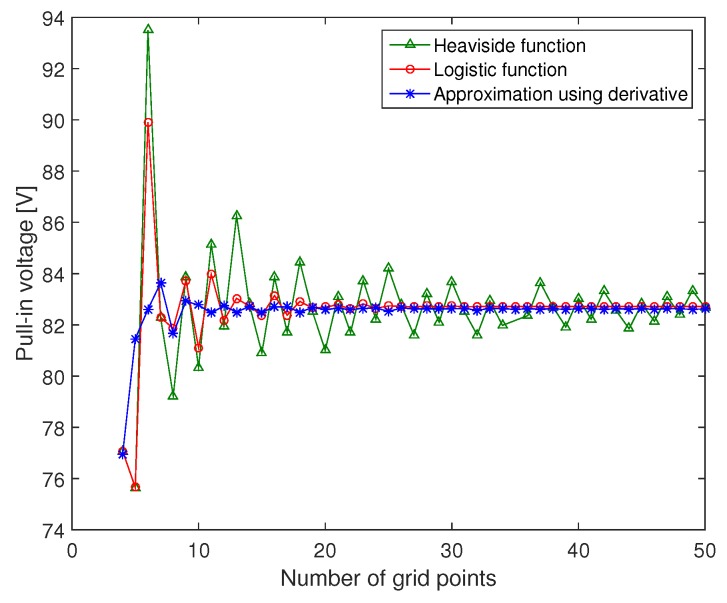
Pull-in voltage of the microplate using the Heaviside function and its approximation with respect to the number of grid points *n*.

In Figure 4, we compare the pull-in voltages between the Heaviside function and its approximation for different number of grid points *n*. For small number of points n≤12, the Heaviside function and the logistic function have the same amplitude and by increasing the number of points the logistic function converges much faster than the Heaviside function. For the second approximation, where we used the derivative of the ramp function, the solution amplitude converges to the same solution as the logistic function. Comparing the three curves, we can conclude that the approximation using the derivative of the ramp function leads to a fast convergence with high accuracy compared to the other functions. For n=10, the static solution of the 3rd approximation converges and the error between the pull-in amplitude for n=10 and n=50 is less than 0.2%.

**Figure 5:**
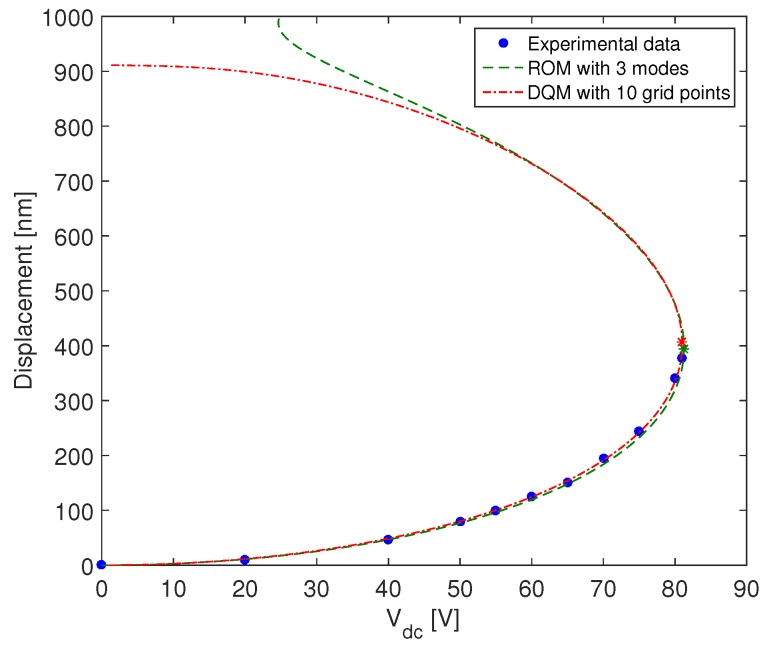
Comparison of the static displacement between the experimental results [47], the reduced order model with three modes and our model with n=10 grid points.

In order to validate the accuracy of the proposed model, we plot the total displacement of the plate at its center with respect to $V_{dc}$ and we compare it to the results presented in [47]. As it is shown in Figure 5, we obtain an excellent agreement between the experimental and the numerical results. For the numerical simulation we used DQM with only n=10 points since 10 points are sufficient to ensure the convergence of the numerical solution. Actually, the static displacement increases monotonously and it is characterized by a vertical slope at the pull-in voltage equal to 81 V and for which the microplate touches the bottom electrode and collapses. Around this excitation level, the plate displacement becomes very sensitive to voltage variations. Clearly, the microplate has no snap-through motion since its static profile shows only one stable mode of operation [18,61,62]. This is because the initial deflection is small compared to the radial dimension of the microplate ($w_{max}<<R$) and the residual stress is negligible. For the lower branch solution, the two analytical models based on DQM and Galerkin method, converge with a few degrees of freedom. However, the use of an even number of modes in the ROM leads to the divergence of the upper branch solution [45].

**Figure 6:**
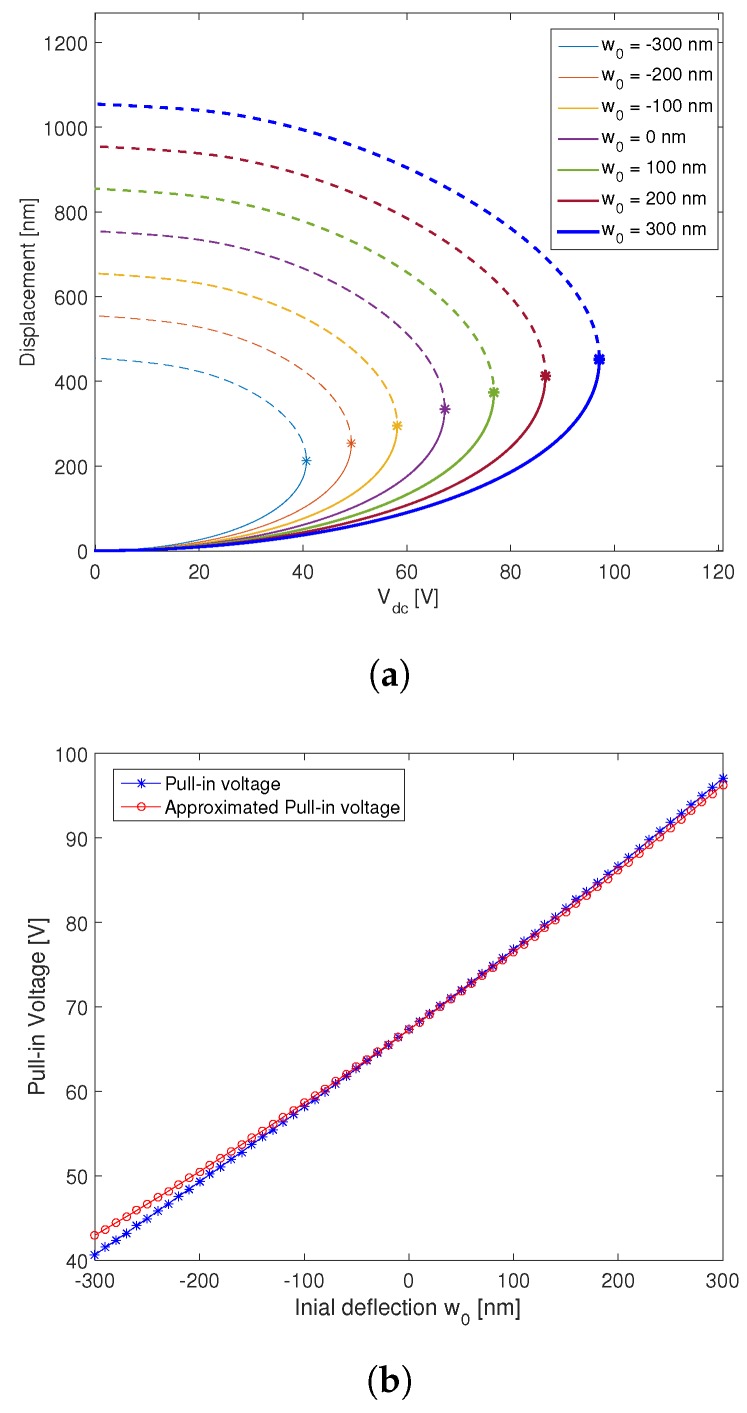
(**a**) The effect of the initial deflection $w_{0}$ on the static response of the microplate. (**b**) Comparison of the pull-in voltage between the proposed and the approximated model.

Then, we investigate theoretically the effect of the initial deflection on the pull-in voltage of circular microplates. Figure 6a displays the variation of the static displacement with respect to the applied DC load for microplates with different initial deflections. The stars (∗) marked in this graph denote the bifurcation points where the solution becomes unstable and goes to pull-in, which increases with respect to the initial deflection. The pull-in voltage increases up to 45% and this is due to the fact that the effective gap between the two electrodes is larger than the expected one d=750 nm, so that a larger electrostatic force is required to reach the pull-in. In order to determine the effective gap distance, we identify the equivalent electrostatic force in the case of initially curved microplates, by integrating over $z\in \left[0,R_{e}\right]$. We obtain the following expression [47]:

(46) $$d_{eff}^{2}\approx \frac{1}{R_{e}}\int_{0}^{R_{e}}{\left(d+w_{0}\left(r\right)\right)}^{2}dr$$

The square of the effective gap distance is approximated as the mean of the square of the total gap distance. In Figure 6b, we compare the pull-in voltage of the circular CMUT calculated using the presented model and approximated using the effective gap distance $d_{eff}$. The proposed approximation has a good agreement with the numerical model especially at small deflections. In our case, the maximum error between the two curves in the interval [−300nm,300nm] is about 5%. Therefore, the effective gap distance is a good approximation to determine the pull-in voltage of a CMUT with initial deflection. However, this approximation is only valid when the initial deflection is small (less than 40% of the gap distance).

## 5. Eigenfrequency Analysis

The initial deflection does not only affect the static response of the microplate, but also it modifies its resonance frequencies. The linear undamped eigenvalue problem of a clamped circular plate excited with a DC voltage is obtained by linearizing (34) and (35) around the static solution. Therefore, we define the total deflection $\left\{w_{i}\left(t\right),u_{i}\left(t\right)\right\}$ as the sum of the static amplitude $\left\{w_{i}^{st},u_{i}^{st}\right\}$ and dynamic component $\left\{w_{i}^{dy}\left(t\right),u_{i}^{dy}\left(t\right)\right\}$. Thus, we write:

(47) $$\begin{array}{cc} w(r=r_{i},t) & =w_{i}\left(t\right)=w_{i}^{st}+w_{i}^{dy}\left(t\right)=w_{i}^{st}+\phi_{i}e^{j\omega t}\\ u(r=r_{i},t) & =u_{i}\left(t\right)=u_{i}^{st}+u_{i}^{dy}\left(t\right)=u_{i}^{st}+\psi_{i}e^{j\omega t} \end{array}$$

where $\left\{\phi_{i},\psi_{i}\right\}$ are the mode shapes of *w* and *u* at $r=r_{i}$. Substituting (47) into Equations (34) and (35), we obtain:

(48) $$\begin{array}{cc} {\ddot{w}}_{i}^{dy}+c{\dot{w}}_{i}^{dy} & +\nabla^{4}\left(w_{i}^{st}+w_{i}^{dy}\right)=N_{0}(\frac{1}{r_{i}}\left(\sum_{j=1}^{n}A_{ij}^{\left(1\right)}\left(w_{j}^{st}+w_{j}^{dy}\right)+w_{0,r}^{i}\right)\\ & +\left(\sum_{j=1}^{n}A_{ij}^{\left(2\right)}\left(w_{j}^{st}+w_{j}^{dy}\right)+w_{0,rr}^{i}\right))+\alpha_{e}\frac{V^{2}\left(t\right)}{{\left(1-\left(w_{j}^{st}+w_{j}^{dy}\right)+w_{0}^{i}\right)}^{2}}\\ & +\gamma {\hat{\Gamma }}_{i}\left(w_{j}^{st}+w_{j}^{dy},u_{j}^{st}+u_{j}^{dy},w_{0}^{i}\right);\;\;i=2,\cdots ,n-2 \end{array}$$

(49) $$\begin{array}{cc} \sum_{j=1}^{n}A_{ij}^{\left(2\right)}\left(u_{j}^{st}+u_{j}^{dy}\right) & +\frac{1}{r_{i}}\sum_{j=1}^{n}A_{ij}^{\left(1\right)}\left(u_{j}^{st}+u_{j}^{dy}\right)-\frac{\left(u_{i}^{st}+u_{i}^{dy}\right)}{r_{i}^{2}}=\\ & -\beta (\frac{1-\nu }{2r_{i}}\left({\left(\sum_{j=1}^{n}A_{ij}^{\left(1\right)}\left(w_{j}^{st}+w_{j}^{dy}\right)\right)}^{2}+2w_{0,r}^{i}\sum_{j=1}^{n}A_{ij}^{\left(1\right)}\left(w_{j}^{st}+w_{j}^{dy}\right)\right)\\ & +\sum_{j=1}^{n}A_{ij}^{\left(2\right)}\left(w_{j}^{st}+w_{j}^{dy}\right)\left(\sum_{j=1}^{n}A_{ij}^{\left(1\right)}\left(w_{j}^{st}+w_{j}^{dy}\right)+w_{0,r}^{i}\right)+w_{0,rr}^{i}\sum_{j=1}^{n}A_{ij}^{\left(1\right)}\left(w_{j}^{st}+w_{j}^{dy}\right));\\ & i=2,\cdots ,n-1 \end{array}$$

The electrostatic force for a circular microplate with initial deflection $w_{\max}$ can be expanded into Taylor series around the static solution:

(50) $$\alpha_{e}\frac{V^{2}\left(t\right)}{{\left(1-\left(w_{i}^{st}+w_{i}^{dy}\right)+w_{0}^{i}\right)}^{2}}=\alpha_{e}\left(V_{dc}+V_{ac}\cos\left(\omega t\right)\right)\left[\frac{1}{{\left(1-w_{i}^{st}+w_{0}^{i}\right)}^{2}}+\frac{2}{{\left(1-w_{i}^{st}+w_{0}^{i}\right)}^{3}}w_{i}^{dy}\right]$$

For n grid points, the total number of equations is equal to 2n. At the boundary nodes, we use the boundary Equation (37) to determine the boundary displacement $\left\{w_{1},w_{n-1},w_{n},u_{1},u_{n-1}\right\}$ as function of $w_{i}$ and $u_{j}$ where i=2,⋯,(n−2) and j=2,⋯,(n−1). This simplification reduces the total number of equations to 2n−5.

The resonance frequencies of the structure and the mode shapes are determined by solving the eigenvalue problem of the system of Equation (48), which can be written as:

(51) $$M\ddot{Y}\left(t\right)+C\dot{Y}\left(t\right)+KY\left(t\right)+K_{NL}\left(Y\left(t\right),\dot{Y}\left(t\right)\right)=0$$

where *M*, *C* and *K* are the mass, damping and stiffness matrices; $K_{NL}\left(Y\left(t\right),\dot{Y}\left(t\right)\right)$ is the vector with nonlinear parameters and Y(t) is the vector of unknowns defined as:

(52) $$Y\left(t\right)=\left\{w_{2}^{d},\cdots ,w_{n-2}^{d},u_{2}^{d},\cdots ,u_{n-1}^{d}\right\}$$

By dropping the nonlinear and damping terms and by replacing $w_{i}^{dy}\left(t\right)=\phi_{i}^{dy}e^{jwt}$ and $u_{i}^{dy}\left(t\right)=\psi_{i}^{dy}e^{jwt}$, the linear natural frequencies are determined using the following characteristic equation.

(53) $$det(K-\omega^{2}M)=0$$

As we can notice in Equation (48), the nondimensional eigenfrequencies depend mainly on the axial force $N_{0}$, the DC voltage $V_{dc}$, the static displacement $w_{i}^{st}$ and the initial deflection $w_{0}$.

**Figure 7:**
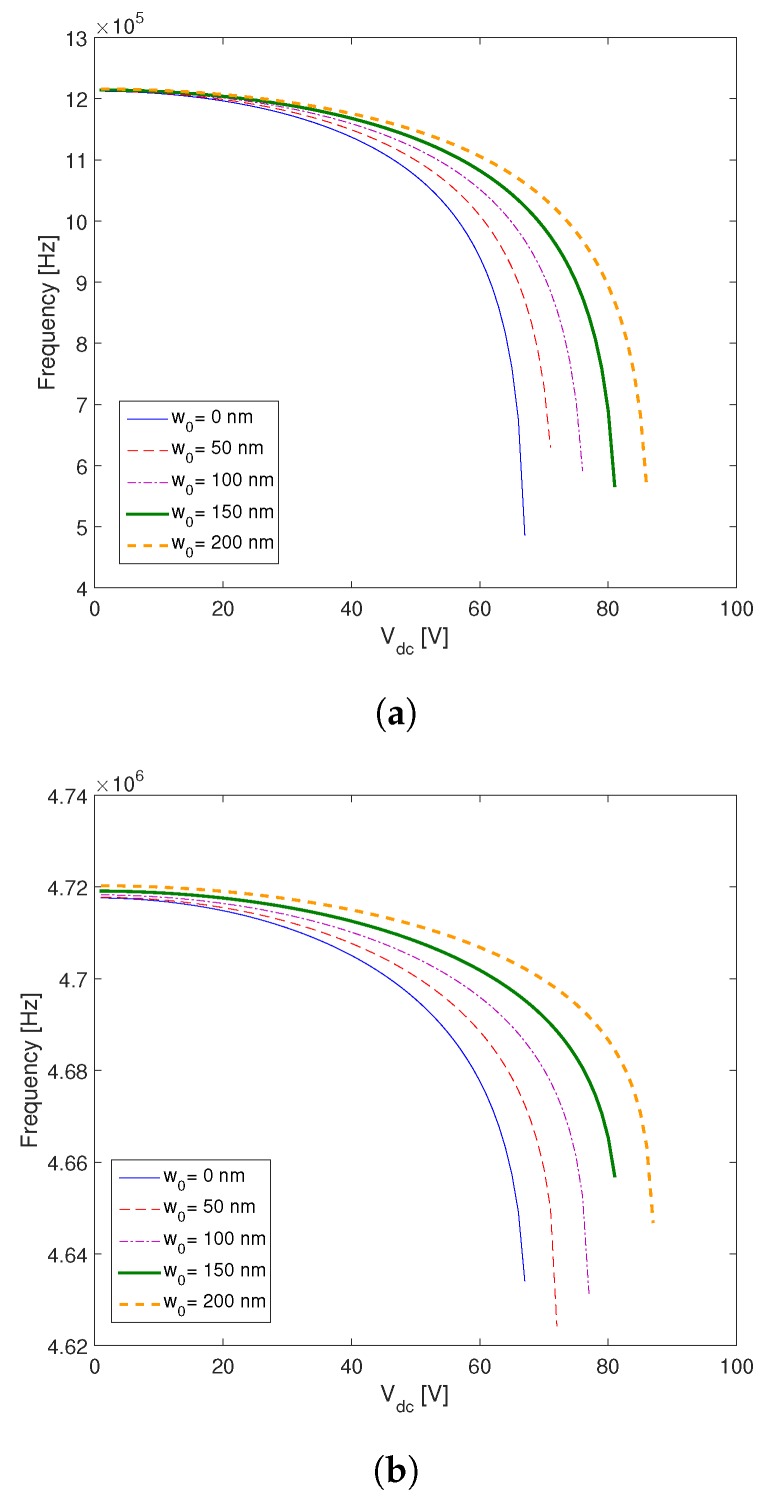
The effect of initial deflection on (**a**) the first and (**b**) the second resonance frequency of the microplate.

As shown in Figure 7, the resonance frequencies decrease as we increase the DC voltage. At the pull in voltage the first resonance frequency drops to zero due to the increase of the electrostatic force. Also, we show the influence of the initial deflection on the first two resonance frequencies. As displayed in Figure 6b, the pull in voltage increases as we increase the initial deflection. At a constant DC voltage, the initial deflection shifts the resonance frequency of the microplate. This is due to the increase of the gap distance which reduces the electrostatic force, i.e., an important actuation voltage is needed to bend the microplate.

## 6. Nonlinear Dynamic Analysis

For *n* grid points, the total number of differential equations to solve is equal to 2n−5, which is increases the computational time. To reduce the number of equations, we write the radial displacement $u_{i}$ as a function of $w_{i}$ using Equation (35). Therefore, Equation (34) can be transformed into:

(54) $$\left[\ddot{X}+C\dot{X}+DX+D_{sq}X_{sq}+D_{cb}X_{cb}\right]\times {\left(g_{d}+X\right)}^{2}=\alpha_{e}V^{2}\left(t\right)$$

where

(55) X=wii=2,⋯,n−2X˙=w˙ii=2,⋯,n−2X¨=w¨ii=2,⋯,n−2Xsq=wiwji,j=2,⋯,n−2i≤jXcb=wiwjwki,j,k=2,⋯,n−2i≤j≤kgd=1−w0ii=2,⋯,n−2

Equation (54) is solved using DQM coupled with the HBM and the ANM, as it is defined in [63,64]. Doing so, one can plot the nonlinear frequency and force responses of the CMUT under primary resonance.

### 6.1. Frequency Response

**Figure 8:**
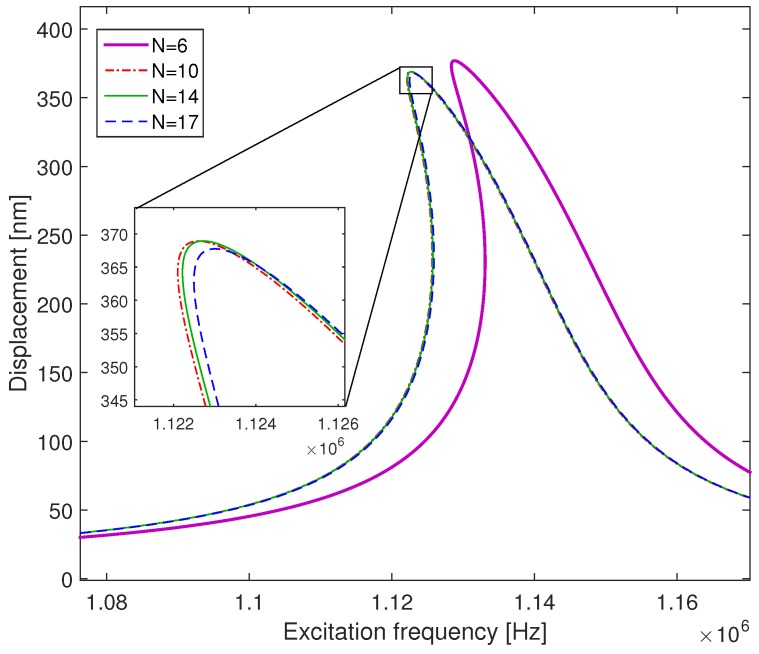
Convergence of the dynamic solution of the microplate actuated with $V_{dc}=50$ V and $V_{ac}=1$ V in the case of a quality factor Q=100.

The frequency response of the microplate is obtained by solving Equation (54) at different excitation frequencies. In order to examine the convergence of the DQM with respect to the number of grid points we simulate the frequency response of a CMUT actuated with a DC voltage $V_{dc}=50$ V and an AC voltage $V_{ac}=1$ V for a quality factor Q=100. In Figure 8, we present the effect of the number of the grid points on the frequency response of the CMUT around its first resonance frequency. The use of a low number of points (n=6) leads to a shift in the resonance frequency and an error in the response of the microplate. By increasing *n*, the nonlinear response of the CMUT converges, and the use of 10 grid points is sufficient to describe the nonlinear dynamic response of the device. The maximum error between the two curves for n=10 and n=17 is less than 1%.

**Figure 9:**
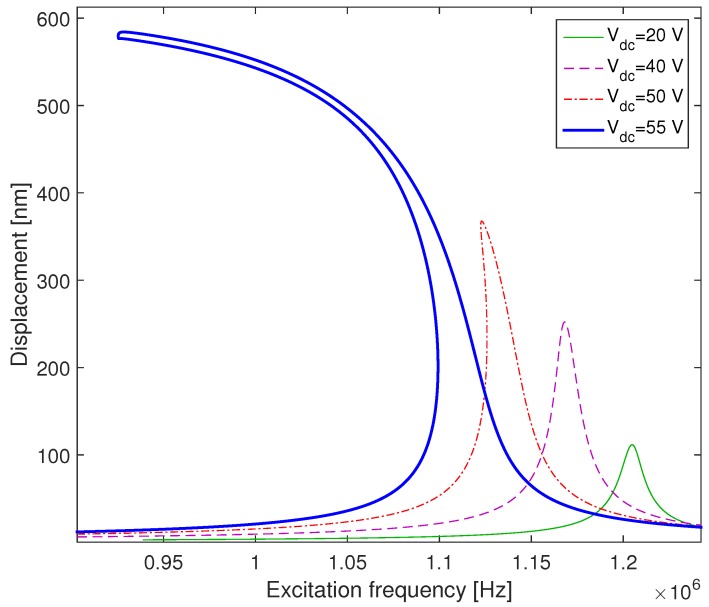
Effects of changing the DC voltage on the frequency responses of the microplate for a $V_{ac}=1$ V and with a quality factor Q=100.

In order to investigate the effects of the actuation voltages, we plot in Figure 9 the frequency response of the CMUT actuated with $V_{ac}=1$ V and for several DC voltages. For a low DC voltage ($V_{dc}=20$ V), the vibration amplitude of the microplate is low and the system exhibits a linear behavior. By increasing the DC voltage, the out-of plane displacement w(r,t) increases and the curve bends to the left and displays a softening behavior due to the increase in the electrostatic nonlinearities. Moreover, the resonance frequency decreases due to the negative stiffness term which is proportional to $V_{dc}^{2}$ and inversely proportional to the cube of the gap distance as it is shown in Equation (50).

**Figure 10:**
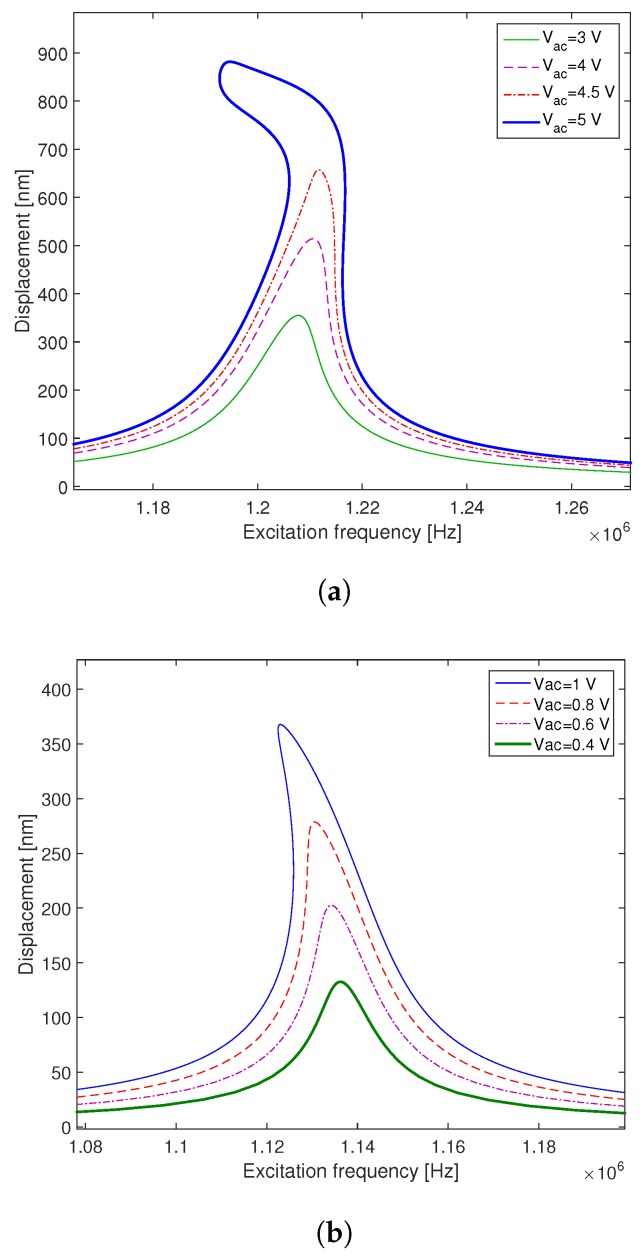
Effects of the AC voltage on the frequency responses of the microplate for: (**a**) a weak DC voltage ($V_{dc}=20$ V; Q=100). (**b**) a high DC voltage ($V_{dc}=50$ V; Q=100).

Unlike the effect of $V_{dc}$ on the CMUT frequency response, Figure 10 shows that the variation of the AC voltage has a significant impact on the excitation amplitude and a negligible one on the negative electrostatic stiffness. Actually, increasing $V_{ac}$ leads to an increase in the vibration amplitude and enlarges the bistability domain without any remarkable frequency shift. This phenomenon is displayed in Figure 10a,b for the case of low and high DC voltage.

In Figure 10a, the CMUT is actuated with a DC voltage $V_{dc}=20$ V. In this case, the increase of the AC voltage from $V_{ac}=3$ V up to $V_{ac}=4$ V leads to an increase in the hardening effect on the nonlinear behavior of the CMUT, while vibrating slightly below the critical amplitude [65,66,67,68]. This is due to the fact that the geometric nonlinearities dominate the microplate dynamics. By increasing more the AC voltage up to ($V_{ac}=5$ V), the CMUT exhibits a mixed behavior [69] due to the increase of the electrostatic force [70], which increases the CMUT vibration up to 80% beyond the critical amplitude.

Remarkably, for $V_{dc}=50$ V (Figure 10b), the increase of the AC voltage leads to a softening behavior beyond the critical amplitude, due to the dominance of the electrostatic nonlinearities over the geometric nonlinearities.

The proposed model enables the capture of the main dynamical phenomena of the CMUT in the frequency domain, which describes the competition between mechanical and electrostatic nonlinearities.

**Figure 11:**
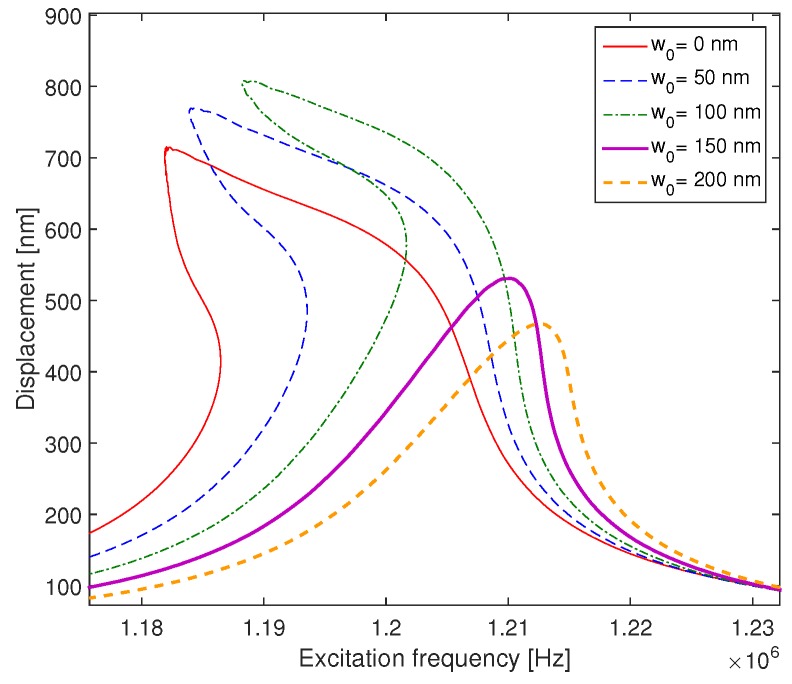
The effect of the initial deflection on the frequency response for the case of microplate excited with $V_{dc}$ = 15 V and $V_{ac}$ = 0.25 V.

In Figure 11, we present the effect of the initial deflection on the frequency response of a microplate actuated with the same AC and DC voltage ($V_{dc}=20$ V and $V_{ac}=4$ V. While a flat microplate has a softening behavior, an increase in the initial deflection leads to a change in the CMUT behavior from softening to hardening. The initial deflection increases the gap distance between the two electrodes, which decreases the electrostatic force. In this case, the geometric nonlinearity becomes larger compared to the electrostatic nonlinearity. Consequently, the frequency response curve bends to the right and exhibits a hardening behavior.

### 6.2. Force Response Analysis

**Figure 12:**
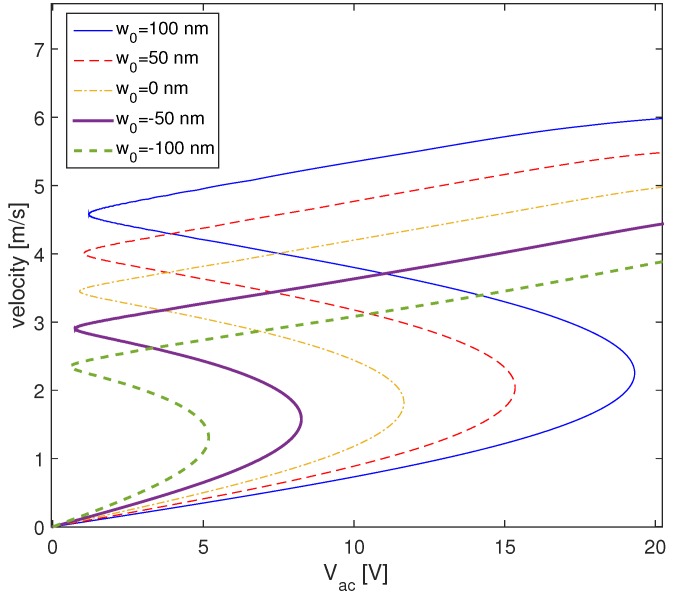
The effect of initial deflection on force response curve, at fixed frequency f=1 MHz and for $V_{dc}=40$ V and Q=500.

For a better understanding of the effect of the excitation amplitude, we display in Figure 12 the force response curves of a circular CMUT with different initial deflections. The force response curves are constructed by varying the excitation voltage $V_{ac}$ while the excitation frequency ω and the bias voltage $V_{dc}$ are set to constant values. For a null AC voltage, the vibration amplitude is equal to zero, because in this case the CMUT is excited only with a DC voltage. For a low $V_{ac}$, the solution amplitude has one possible solution. By raising the AC voltage, the vibration amplitude increases and the dynamic solution becomes nonlinear and displays bistability.

In Figure 12, we display the effect of an initial deflection on the force response curve in the case of a softening behavior. It is shown that as the initial deflection increases, the bifurcation point is shifted to the right, which enlarges the bistability domain. This is due to the increase in the effective gap distance, which means that a higher AC voltage is needed to reach the bifurcation point.

## 7. Conclusions

In this paper, the mathematical model of a circular CMUT has been derived while taking into account the effects of an axisymmetric initial deflection and the main sources of nonlinearities. The partial differential equations have been spatially discretized using the DQM. The resulting equations are then transformed into coupled ODEs. For the CMUT static behavior, we presented the good agreement between the experimental results and the numerical solutions, which proves that DQM, with only 10 grid points, is an efficient tool to study the static response of such a device. We showed that a positive initial imperfection increases the pull-in voltage of the CMUT. Also, we presented an approximation of the effective gap distance that can be used with a flat plate model to predict the pull-in voltage.

The numerical model has been also used to investigate the effect of the initial deflection on the resonance frequencies of a CMUT at several DC voltages. For the nonlinear frequency responses, the ODEs have been solved using the HBM coupled with the ANM. It has been shown that the CMUT can exhibit a hardening, a softening or a mixed behavior depending on the forcing parameter $V_{dc}$. Also, we have highlighted that the initial deflection can change the frequency response of the CMUT from softening to hardening, by increasing the effective gap distance, which decreases the electrostatic nonlinearities. Finally, we have shown that the initial deflection shifts the bifurcation points to the right while enlarging the bistability domain.

Future work will include the investigation of the effects of the squeezed air film and the fluidic medium on the nonlinear response of the microplate.

## Figures and Tables

**Table 1:** Physical parameters of the CMUT.

Symbol	Quantity	Dimension
*E*	Young’s modulus	149 [GPa]
ρ	Density	2330 [kg/m^3^]
ν	Poisson’s ratio	0.27
*R*	Radius of the microplate	86 [µm]
$R_{ele}$	Radius of the electrode	54 [µm]
*h*	Thickness of the microplate	2.3 [µm]
*d*	Gap distance	0.75 [µm]
$w_{0}$	Initial deflection	161 [nm]

## Appendix A

The continuation method is used to determine how the solution of a system varies with respect to a certain parameter. We consider the following algebraic system:

(A1) F(X,α)=0

where α is the continuation parameter and *X* is the unknown parameter $X\in R^{n}$. The algebraic system (A1) can have many branches for the same parameter α. Therefore, the continuation method is used to follow the path of a solution branch at an initial parameter $\alpha_{0}$. The determination of all the initial solution $X_{0}$ can be done using a homotophy algorithms [71,72,73].

In order to solve this algebraic system (A1) we use the arclength continuation technique to determine how the solution changes with respect to α. The idea of this method is to avoid varying the parameter α by parametrizing the variables *X* and α via a new variable *s*, X=X(s) and α=α(s). The system of equations becomes:

(A2) F(X(s),α(s))=0

To solve the system of equations we add an additional equation specified by Euclidean arclength normalization [74].

(A3) $$X^{T}X+\alpha^{2}=1$$

where the initial conditions for (A2) and (A3) are given by

(A4) $$\begin{array}{cccccccccccccccc} X & =X_{0} & & & \mathrm{and} & & & \alpha & =\alpha_{0} & & & \mathrm{at} & & & s & =0 \end{array}$$

Compared to the sequential continuation method, the arclength method is more automatic in determining the solution branches because this method follows the branches of the solution which makes it possible to determine the branches in the case of the turning point.

References
